# Impact of Dietary *Moringa oleifera* Leaf Polysaccharide on Growth Performance and Antioxidant Status in Broiler Chickens

**DOI:** 10.3390/vetsci12121196

**Published:** 2025-12-13

**Authors:** Hosameldeen Mohamed Husien, WeiLong Peng, Raza Mohai Ud Din, Mudathir Yahia Abdulrahman, Nada N. A. M. Hassanine, Mohamed Osman Abdalrahem Essa, Saber Y. Adam, Hozifa S. Yousif, Ahmed A. Saleh, Mengzhi Wang, Jingui Li

**Affiliations:** 1 College of Veterinary Medicine, Yangzhou University, Yangzhou 225009, China; 008643@yzu.edu.cn (H.M.H.); wlpeng98@163.com (W.P.); mohosman0999@gmail.com (M.O.A.E.); 2 College of Animal Science and Technology, Yangzhou University, Yangzhou 225009, China; mh24136@stu.yzu.edu.cn (R.M.U.D.); mudathir@duc.edu.sd (M.Y.A.); nedo1.maro2@gmail.com (N.N.A.M.H.); saber@duc.edu.sd (S.Y.A.); elemlak1339@yzu.edu.cn (A.A.S.); 3 College of Veterinary Medicine, Albutana University, Rufaa 22217, Sudan; hozifaedu2@albutana.edu.sd; 4 Animal and Fish Production Department, Faculty of Agriculture (Al-Shatby), Alexandria University, Alexandria City 11865, Egypt; 5 State Key-Laboratory of Sheep Genetic Improvement and Healthy-Production, Xinjiang Academy of Agricultural Reclamation Sciences, Shihezi 832000, China

**Keywords:** MOLP, natural product, broiler chickens, growth performance, antioxidant indices

## Abstract

*Moringa oleifera* (MO) is a versatile non-traditional feed supplement rich in bioactive compounds. The objective of this study was to examine the effects of MO leaf (MOL) polysaccharide (MOLP) intake as a natural product on broiler chicken production and antioxidant indices. A polysaccharide with a molecular weight of 182.989 kDa was isolated from MOL in a previous study. Broiler chickens were allocated at random into four groups receiving varying doses of MOLP (0, 0.1, 0.2 and 0.4 g/kg feed) for three weeks. Feed intake (FI), average daily feed ingestion (ADFI), feed conversion ratio (FCR), and body weight gain (BWG) were monitored. Serological markers, including total protein (TP), albumin (ALB), globulin (GLO), albumin-to-globulin ratio (ALB/GLO), creatinine (CREA), as well as the activities of total superoxide dismutase (T-SOD), glutathione peroxidase (GSH-Px), total antioxidant capacity (T-AOC) and the concentrations of malondialdehyde (MDA) were assessed. Results from days 21 to 28 demonstrated that the high dose of MOLP significantly enhanced BWG, ADFI, liver and bursa indices compared to the control group. Additionally, TP and GLO, T-SOD, GSH-Px, T-AOC and MDA levels were elevated (*p* < 0.05). In conclusion, MOLP supplementation, particularly at 0.4 g/kg feed, positively impacted broiler chicken growth performance and antioxidant indices, suggesting its potential as a valuable feed additive.

## Simple Summary

This study tested Moringa oleifera leaf polysaccharide (MOLP) as a feed additive for broilers. Supplementation at 0.4 g/kg feed improved final body weight and feed intake in later growth stages. It also boosted the body’s antioxidant defenses, increasing key enzyme activities (T-SOD, GSH-Px, T-AOC) and reducing oxidative stress (MDA). Positive effects on physiological indicators were noted, such as increased organ weights and serum protein levels at specific stages. The results highlight MOLP, particularly at 0.4 g/kg, as a promising natural additive for enhancing broiler productivity and health.

## 1. Introduction

The poultry trade has experienced extraordinary expansion over the past two decades, becoming a critical contributor to global protein security [1]. It plays a vital role in bridging the gap between the rising demand for high-quality protein for human consumption and its availability. However, this rapid growth has been accompanied by significant challenges related to poultry health. Infectious diseases caused by viruses, bacteria, fungi, and protozoa pose a major threat to poultry production, leading to substantial economic losses [2].

Traditionally, the poultry industry has relied heavily on medications for treatment and protective purposes [1]. While this approach initially helped control infectious diseases, it has culminated in the unintended consequence of antibiotic residues accumulating in chicken meat and eggs [2]. These residues pose a potential health risk to human consumers, as they can contribute to the development of antimicrobial resistance in gut microbiota and pathogenic microbes [3]. Furthermore, the extensive utilization of medicines in chickens may lead to cross-resistance, where bacteria become resistant to antibiotics used in both human and animal medicine, thereby compromising their therapeutic effectiveness [4].

*Moringa oleifera* (MO) is affiliated with the Moringaceae family and is native to South Asia, southwestern Africa, Madagascar, and Arabia [5]. Owing to its rich content of phenolic compounds and polysaccharides [6], MO has gained prominence as a valuable nutraceutical and therapeutic plant [7]. The most utilized part is the MO leaf (MOL) [8]. The bioactive compounds in MO offer potential for developing functional foods with enhanced nutritional and health benefits [9].

Plant-derived bioactive components, particularly polysaccharides, have received significant interest for their diverse biological activities, with research suggesting they could support immune system functioning and gut health in poultry [10,11,12]. For example, the polysaccharide from *Acanthopanax senticosus* (ASP) has been demonstrated to enhance immune responses and possesses antioxidant and anti-inflammatory properties [13,14,15]. Similarly, *Lycium barbarum* polysaccharide (LBP) has been shown to modulate serum cytokine levels [16]. In poultry, dietary MOL has been associated with increased serum protein levels and improved protein metabolism [17,18,19]. The antioxidant properties of MOL components are considered a fundamental mechanism that may underpin these observed health benefits [20,21].

Based on the documented bioactive properties of *M. oleifera* polysaccharides, this study was designed to evaluate the effects of MOLP supplementation on broiler growth performance and systemic antioxidant capacity. Production metrics, serum antioxidant enzyme activities, and key physiological indicators including organ development and serum biochemistry were assessed to determine its efficacy as a natural feed additive.

## 2. Materials and Methods

### 2.1. Preparation and Extraction of Polysaccharide from MOL

**Figure 1:**
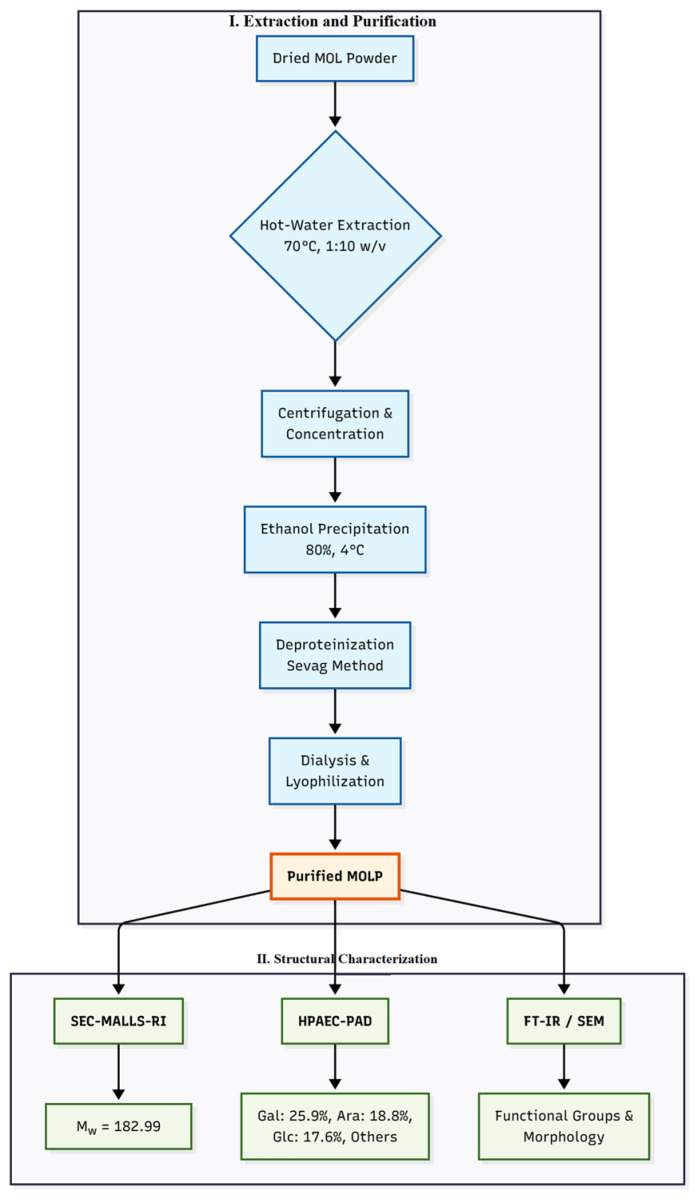
Schematic workflow for the isolation and structural analysis of *Moringa oleifera* leaf polysaccharide (MOLP). The process comprises two sequential phases. Phase I (Extraction & Purification): Dried leaf powder was subjected to hot-water extraction, ethanol precipitation, deproteinization (Sevag method), dialysis, and lyophilization to obtain purified MOLP. Phase II (Structural Characterization): The final product was analyzed to determine its molecular weight (Mw = 182.99 kDa) by SEC-MALLS-RI, its monosaccharide composition (galactose, arabinose, and glucose as major sugars) by HPAEC-PAD, and its structural features by FT-IR spectroscopy and SEM.

Dried MOLP, commercially sourced from Yunnan Honglv Biotechnology Co., Ltd. (Kunming, Yunnan, China), served as the raw material. The crude polysaccharide was extracted using a standardized hot-water extraction method. Briefly, MOL powder was mixed with deionized water (Milli-Q, Millipore, Burlington, MA, USA) at a solid-to-liquid ratio of 1:10 (*w*/*v*) and extracted at 70 °C for 90 min under constant agitation. This extraction process was repeated three times to maximize yield. The combined aqueous extracts were centrifuged at 4000 rpm for 20 min (5810R refrigerated centrifuge, Eppendorf, Hamburg, Germany) to remove insoluble particulates. The collected supernatants were then concentrated using a rotary evaporator (RV 10, IKA, Staufen, Germany) at reduced pressure. Polysaccharides were precipitated from the concentrated solution by adding chilled ethanol (≥99.8%, Sigma-Aldrich, St. Louis, MO, USA) to a final concentration of 80% (*v*/*v*) and incubating overnight at 4 °C. The resulting precipitate was recovered by centrifugation (5810R, Eppendorf, Hamburg, Germany), washed successively with 95% ethanol (Sigma-Aldrich, St. Louis, MO, USA), and then re-dissolved in deionized water. To remove protein contaminants, the aqueous polysaccharide solution was subjected to deproteinization using the Sevag method (chloroform ≥ 99.8%, Sigma-Aldrich, St. Louis, MO, USA; n-butanol ≥ 99.5%, Sigma-Aldrich, St. Louis, MO, USA; mixed at 4:1 *v*/*v*), repeated until no proteinaceous interface was observed. The purified aqueous phase was transferred into dialysis tubing (Spectra/Por^®^ 3.5 kDa MWCO dialysis membrane, Spectrum Laboratories, Rancho Dominguez, CA, USA) against distilled water for 48 h and finally lyophilized to obtain the purified MOLP as a light-yellow powder, which was stored desiccated until use [6,22], (Figure 1).

### 2.2. Structural Evaluation of MOLP

The isolated MOLP was subjected to comprehensive structural analysis. The weight-average molecular weight (Mw), determined by Size-Exclusion Chromatography coupled with Multi-Angle Laser Light Scattering and Refractive Index detection (SEC-MALLS-RI), was 182.989 kDa. Analysis was performed using a DAWN HELEOS-II laser photometer (Wyatt Technology, Santa Barbara, CA, USA) equipped with tandem Shodex OH-pak SB-800 series columns, eluted with 0.1 M NaNO_3_ containing 0.02% NaN_3_ at a flow rate of 0.4 mL/min at 45 °C.

Monosaccharide composition analysis by High-Performance Anion-Exchange Chromatography with Pulsed Amperometric Detection (HPAEC-PAD) revealed MOLP to be a heteropolysaccharide. The predominant neutral sugars were galactose (25.90%), arabinose (18.81%), and glucose (17.57%), which collectively constituted over 60% of the composition. Other components included rhamnose (12.04%), xylose (12.01%), mannose (3.51%), galacturonic acid (5.28%), and minor amounts of fucose, glucuronic acid, and mannuronic acid. For this analysis, MOLP was hydrolyzed with 2 M trifluoroacetic acid (TFA) at 121 °C for 2 h, and the hydrolysate was analyzed on a CarboPac PA20 column using a gradient elution of NaOH and NaOAc.

Further structural insights, including Fourier-transform infrared (FT-IR) spectroscopy confirming typical polysaccharide functional groups and scanning electron microscopy (SEM) revealing its morphological features, are consistent with our detailed prior report [6] and confirm the successful isolation of the intended bioactive polysaccharide (Figure 1).

### 2.3. Experimental Design

A total of 120 female Chinese yellow broiler chicks, 7 days old with an initial body weight (BW) of 61.42 ± 1.39 g, were obtained from the farm of Yangzhou University. Upon arrival, the chicks were reared under standard conditions for a 7-day acclimatization period. At 14 days of age, which was designated as Day 0 of the experiment, the following procedures commenced.

Experimental animals were randomly divided into four groups with 3 replicates per treatment, with 10 broilers per replicate. The experimental unit was the replicate pen. The number of replicates per treatment (n = 3) was determined based on common practice in poultry nutrition research and represents a standard design capable of detecting significant differences in growth performance parameters while accounting for practical constraints related to animal housing and resource allocation. The control group received a basal diet, while the experimental groups were fed the same diet supplemented with MOLP at three dosage levels: 0.1, 0.2, and 0.4 g/kg of feed (MOLP-L, MOLP-M, and MOLP-H, respectively). The supplementation period lasted for 21 days (from Experimental Day 0 to Day 20).

The experimental diets were formulated by supplementing the basal diet with MOLP at the expense of rape seed cake on a weight-to-weight basis to maintain isocaloric and isonitrogenous conditions. Consequently, as the MOLP inclusion level increased from 0 to 0.4 g/kg, the rape seed cake content decreased proportionally from 9.00% to 4.00% (Table 1). The minor corresponding adjustments in corn and soybean meal were made to account for this substitution and maintain consistent calculated metabolizable energy and crude protein levels across all diets. The observed minor variations in the analyzed crude protein content (Table 1) are within the margin of analytical error for feed formulation and are not considered to have a biologically significant impact on the study outcomes.

Growth performance and organ indices were evaluated over three phases: the starter phase (Experimental Days 0–7; birds aged 14–21 d), grower phase (Experimental Days 8–14; birds aged 22–28 d), and finisher phase (Experimental Days 15–20; birds aged 29–35 d). The basal and experimental diet formulations are detailed in Table 1.

The details of the experiment are as follows: During the experimental period, standard disinfection procedures were followed. The temperature in the brooder was gradually decreased from 35 °C to a constant 27 °C. A lighting program of 23 h of light and 1 h of darkness (23L:1D) was maintained throughout the trial. Relative humidity was maintained at 60%. Birds were housed in floor pens with a stocking density of 10 birds per pen, equivalent to 10 birds/m^2^ (based on an assumed pen size of 1 m^2^). They were provided ad libitum access to feed (provided as mash) and water. Any bird showing severe signs of illness or injury unrelated to the treatment was removed from the study and excluded from data analysis; mortality and the reasons for any exclusions were recorded. Conventional practices were adhered to for vaccinations, sanitation, and disinfection of the experimental chickens.

Feed was provided twice daily, at 8:00 a.m. and 4:00 p.m., allowing the broilers unlimited access to food and water. Body weight and feed intake were recorded for all birds at the beginning and end of each growth phase (Experimental Days 0, 7, 14, and 20) to calculate performance parameters for the starter, grower, and finisher periods. For serum biochemistry and antioxidant analysis, a cross-sectional sampling design was employed. At the conclusion of each phase (Days 7, 14, and 20), blood samples were collected from the jugular vein of two randomly selected birds per replicate (n = 6 per treatment per time point); these birds were then removed from the study. Organs (liver, spleen, and bursa of Fabricius) were collected and weighed only during the terminal slaughter on Experimental Day 20 from all remaining birds.

### 2.4. The Average Daily Feed Intake (ADFI) and the Ratio of Feed Conversion (FCR)

The following standard formulas were used to calculate growth performance parameters for each phase and overall:

**Average Daily Feed Intake (ADFI, g/bird/day)** = Total feed intake per pen (g)/(Number of birds per pen × Number of days in the period)
**Average Daily Gain (ADG, g/bird/day)** = (Final body weight − Initial body weight) (g)/Number of days in the period
**Feed Conversion Ratio (FCR)** = Total feed intake per pen (g)/Total body weight gain (BWG) per pen (g).

FCR is expressed as g of feed per g of gain.

### 2.5. Samples of Blood and Contents Obtaining

Blood samples were drawn from the jugular vein and centrifuged at 2000 rpm per minute for 10 min. The serum was subsequently stored at −20 °C for further analysis. Following euthanasia, organs (liver, spleen, and bursa) were carefully removed, divided into smaller sections, and placed in microtubes within a sample box containing liquid nitrogen. All samples were stored at −80 °C for Further use. The levels of serum total protein (TP), albumin (ALB), globulin (GLO), albumin-to-globulin ratio (ALB/GLO), and creatinine (CREA) were analyzed. The activities of total superoxide dismutase (T-SOD), glutathione peroxidase (GSH-Px), total antioxidant capacity (T-AOC), and the concentrations of malondialdehyde (MDA) were measured.

### 2.6. Statistical Analysis

All data are expressed as the mean ± standard error of the mean (SEM). The experimental unit was the pen. To prevent pseudoreplication, data for all parameters from individual birds within a pen were averaged to yield one value per replicate. Statistical analysis was performed on these pen means using one-way ANOVA followed by Duncan’s multiple range test in IBM SPSS 22.0 software. Differences were considered statistically significant at *p* < 0.05.

## 3. Results

### 3.1. Effect of MOLP on the Growth Performance

The effects of MOLP on growth performance across different phases are presented in Table 2. During the initial phase (days 0–14), no significant differences (*p* > 0.05) were observed in BWG, ADFI, or average daily gain (ADG) among the dietary groups.

In the grower phase (days 15–21), supplementation with MOLP at 0.4 g/kg (MOLP-H) significantly increased the final average body weight to 244.72 ± 9.28 g compared to 210.61 ± 5.81 g in the control group (*p* < 0.05). This was accompanied by a higher average daily feed intake (59.01 ± 2.65 g/day vs. 55.00 ± 2.14 g/day) and ADG (29.59 ± 1.90 g/day vs. 26.19 ± 1.50 g/day) in the MOLP-H group relative to the control.

These positive effects persisted into the finisher phase (days 22–28). The average body weight of the MOLP-H group (345.90 ± 15.18 g) remained significantly greater than that of the control group (280.04 ± 17.83 g). Similarly, the ADG for MOLP-H was 35.77 ± 1.87 g/day, which was significantly higher than the 31.92 ± 1.94 g/day observed in the control. FCR showed a numerical improvement in MOLP groups, particularly for MOLP-H (2.26 ± 0.92 vs. 2.81 ± 0.64 for the control), although this difference did not reach statistical significance (*p* > 0.05) among the treatment groups.

### 3.2. Effect of MOLP on the Proportional Weight of Organs

The effects on organ indices are presented in Table 3. From days 14 to 21, slight variations were observed in bursa and spleen weights within the treated groups, but these changes were not statistically significant (*p* > 0.05). However, on day 14, the MOLP-H group exhibited a significant increase in liver weight (*p* < 0.05) compared to the control. Additionally, on day 28, the MOLP-H group demonstrated a significant increase in bursa weight (*p* < 0.05) relative to the control. The differences in the relative weights of the spleens in all MOLP treatment groups were not statistically significant (*p* > 0.05) in comparison to the control.

### 3.3. Effect of MOLP on Blood Biochemical Analysis

Serum TP and GLO levels exhibited minor fluctuations across the MOLP groups with increasing doses. These alterations were not of statistical significance (*p* > 0.05) on day 14. From days 21 to 28, TP and GLO levels in the MOLP-H group significantly increased (*p* < 0.05). Moreover, from days 14 to 28, the levels of ALB, LB/GLO, and CREA within each MOLP group remained statistically similar to the control group (*p* > 0.05) as shown in Table 4.

### 3.4. The Contents of Serum Antioxidants

Table 5 shows that T-SOD, GSH-Px, T-AOC and MDA levels exhibited minor fluctuations across the MOLP groups with increasing doses. These alterations were not of statistical significance (*p* > 0.05) on days 14 and 21. However, from days 22 to 28, MOLP groups showed a significant increase (*p* < 0.05) in the activities of T-SOD, GSH-Px, and T-AOC, while MDA levels significantly reduced (*p* < 0.05) compared with the control group.

## 4. Discussion

The present investigation found that dietary MOLP supplementation influenced broiler chickens. High-dose MOLP showed modest improvements in BW and ADFI in late growth stages and improved antioxidant indices at 22–28 d. These observed improvements suggest the efficacy of MOLP as a beneficial feed additive.

Our results on growth performance are comparable with those revealed by Khan et al. [23], who found that MOL powder supplementation influences growth performance and gut morphology in broilers. Specifically, our high-dose MOLP group (0.4 g/kg feed) achieved a final body weight of 345.90 ± 15.18 g, representing a 23.5% increase over the control group (280.04 ± 17.83 g) during the finisher phase (*p* < 0.05, Table 2). It is noteworthy that while Khan et al. [23] used a 5–10% inclusion of crude leaf powder, our study demonstrates that a much lower concentration (0.04%) of the purified polysaccharide fraction is efficacious. The improvement in feed efficiency may be attributed to the nutritional properties of MOLP, particularly its carbohydrate content, including dietary fibers. Similarly, studies involving *Aspergillus oryzae* (AO) have reported a significant enhancement in broiler body weight when supplemented [24,25,26].

The significant increase in bursa weight observed in the MOLP-H group on day 28 (1.94 ± 0.23% vs. 1.38 ± 0.20% in controls, *p* < 0.05, Table 3) is consistent with other studies on the relative weights of bursa in MOL meal diets [25]. Furthermore, the MOLP-H group exhibited a significant increase in relative liver weight on day 14 (5.40 ± 0.42% vs. 4.56 ± 0.46%, *p* < 0.05). These changes in key metabolic and organs are physiologically linked to the potent antioxidant activity of MOLP. The findings from the blood biochemical analysis, including elevated total protein and globulin, suggest that MOLP supplementation supports a positive physiological and health status in chickens. Within the specific parameters measured in this study growth performance, serum biochemistry, antioxidant status, and organ indices MOLP supplementation did not elicit any negative responses. This observation aligns with the results of Melesse et al. [19], who reported that the inclusion of MO meal in poultry diets significantly enhanced various serum biochemical characteristics.

The findings on serum antioxidants imply that MOLP has beneficial effects on broiler chickens’ antioxidant protection function. This is comparable to Song et al. [27], who found that increasing the amount of MOL powder in meals improved serum T-SOD, GSH-Px and T-AOC while reducing MDA levels. In our study, the MOLP-H group showed a significant reduction in serum MDA (6.46 ± 0.42 nmol/mL vs. 7.28 ± 0.27 nmol/mL in controls) and elevated T-AOC and GSH-Px activities (*p* < 0.05, Table 5). Previous research has also shown that dietary supplementation with MOLP can increase tissue SOD activities while decreasing hepatic MDA levels in Nile tilapia [28]. The increased antioxidant capacity could be attributed to chlorogenic acid, quercetin, and vitamin E in MOLP, all of which have strong free radical scavenging activities and reduce lipid peroxidation [29,30]. We propose that this antioxidant action protects hepatocytes, supporting the observed increase in liver weight and its metabolic functions, including the synthesis of proteins.

This enhanced hepatic function is reflected in the serum biochemistry. The MOLP-H group showed significantly higher levels of total protein (43.60 ± 0.50 g/L) and globulin (26.23 ± 0.53 g/L) compared to the control (34.93 ± 2.02 and 18.63 ± 0.46 g/L, respectively) during the grower phase (Table 4). This indicates an improved anabolic status and protein metabolism, directly contributing to the measured weight gain.

These findings align with the broader body of research exploring various natural feed additives in poultry diets. The results of this study resonate with Tran et al. [31], who found that *Prinsepiae nux* extract supplemented diets significantly improved FCR and hepatic expression of antioxidant genes. Similarly, the study by Chen et al. [32] corroborates our findings, showing that a complex antioxidant blend significantly improved broiler growth performance and antioxidant markers. Yang et al. [33] demonstrated that a probiotic effectively promotes broiler growth and boosts antioxidant indices, suggesting that MOLP might serve as an efficient natural alternative.

Studies by Wu et al. [34] and Soren et al. [35] illustrated that dietary lycopene and probiotics/postbiotics supplementation enhance growth performance and gut health. Furthermore, the study by Chen et al. [36] on *Zanthoxylum bungeanum* seed meal found similar improvements in growth and antioxidant capacity. The positive impact on BWG and feed efficiency observed with nanoencapsulated rosemary essential oil [37] and other natural supplements like rosemary leaf powder [38], *Chlorella vulgaris* [39], and methionine [40] correlates well with this study’s findings on MOLP, suggesting similar mechanisms of action.

Additionally, Zhang et al. [41] investigated the effects of using MOL as a protein feed substitute and reported that a 5% inclusion level significantly enhanced growth performance and carcass quality, which corroborates our results achieved with 0.04% purified MOLP. It is acknowledged that in our study, MOLP was included at the partial expense of rape seed cake. While the primary effects are attributed to MOLP, the potential for interactive effects between the bioactive compounds of MOLP and those present in rape seed cake (e.g., glucosinolates) on nutrient digestibility or gut microbiota cannot be ruled out and presents a valuable avenue for future research. These positive effects on physiological and health parameters associated with MOLP supplementation are consistent with the broader benefits of natural feed additives.

Briefly, our findings, together with various studies on natural feed additives, converge on a pivotal theme: natural compounds like MOLP can effectively boost growth performance, antioxidant capacity, and overall health in broiler chickens. These improvements are critical for sustainable poultry production and reducing dependence on traditional antibiotics and synthetic additives.

While this study provides clear evidence for the efficacy of MOLP as a feed additive, several considerations merit attention to guide future research. First, it should be noted that MOLP was included in the diet at the partial expense of rape seed cake, which contains its own bioactive compounds such as glucosinolates. Although the primary effects are attributed to MOLP, the potential for interactive effects between these components on nutrient digestibility, antioxidant status, or gut microbiota cannot be ruled out and presents a valuable avenue for mechanistic investigation. Second, to fully establish its safety profile, comprehensive toxicological assessments are warranted, including detailed mortality and clinical records, histopathological examinations, and an extended panel of hepatic and renal function markers. Finally, applied research should investigate the optimal timing and duration of supplementation, effects on meat quality, and efficacy under commercial-scale farming conditions.

## 5. Conclusions

The results of this study indicate a threshold effect, with the significant benefits on growth and antioxidant status being achieved specifically at the 0.4 g/kg supplementation level, which we identify as the most effective dose tested in this study. This concentration significantly improved BWG and ADFI between days 14 and 28 and supported physiological development, as indicated by increased liver and bursa weights. The high MOLP dose also elevated serum TP and GLO levels and enhanced the activities of T-SOD, GSH-Px, and T-AOC. Under the present experimental conditions, a clear dose-dependent response was observed, with 0.4 g/kg identified as the optimal recommended dose. Further research is warranted to validate these findings under commercial production settings and to explore additional potential effects of MOLP on poultry performance.

## Figures and Tables

**Table 1:** Ingredient (%) and nutritive value of the basic diet and experimental diet.

Items	Control	MOLP-L	MOLP-M	MOLP-H
Ingredients %				
Corn	61.3	61.2	60.9	60.6
MOLP	00.0	0.10	0.20	0.40
Soybean meal	21.1	21.2	21.5	21.8
Rape seed cake	9.00	8.00	6.00	4.00
Wheat bran	4.50	4.50	4.50	4.50
NaCl	0.500	0.500	0.500	0.500
Limestone powder	1.40	1.40	1.40	1.40
Calcium hydrogen phosphate	1.80	1.80	1.80	1.80
Lysine (Lys)	0.100	0.100	0.100	0.100
DL-Met	0.100	0.100	0.100	0.100
Mineral premix	0.0700	0.0700	0.0700	0.0700
Vitamin premix *	0.0300	0.0300	0.0300	0.0300
Choline chloride	0.800	0.800	0.800	0.800
Total Nutrient composition	100	100	100	100
Crude protein (CP %)	18.2	18.1	18.1	18.2
Metabolizable energy (ME) (MJ/kg)	11.7	11.7	11.7	11.7
Lysine (Lys) + methionine (Met) (%)	0.760	0.770	0.770	0.760
Calcium (Ca) ^†^ (%)	0.900	0.900	0.900	0.900
Phosphorus ^†^ (P) (%)	0.440	0.440	0.440	0.440

Nutrient composition calculations: The crude protein (CP, N × 6.25), metabolizable energy (ME), amino acids (Lys, Met), calcium (Ca), and phosphorus (P) contents presented are calculated values based on the ingredient composition and their respective nutritional profiles from standard feed tables (e.g., Chinese Feed Database). ME was calculated using the predictive equation for poultry. The analyzed CP values are also provided for verification. * Provided vitamins per kg of the feed; ^†^ Supplied minerals per kg of the feed.

**Table 2:** Effects of MOLP supplementation on body weight gain (BWG), average daily feed intake (ADFI), average daily gain (ADG), feed conversion ratio (FCR), and feed-to-gain ratio (F/G) of broiler chickens at different growth phases (0–14, 15–21, and 22–28 days).

Groups					
Items	Control	MOLP-L (0.1 g/kg)	MOLP-M (0.2 g/kg)	MOLP-H (0.4 g/kg)	*p*-Value
0–14 d					
BWG (g/d)	149.02 ± 6.20	148.29 ± 6.01	153.94 ± 6.02	146.28 ± 4.57	0.456
ADFI (g/d)	42.84 ± 1.12	44.74 ± 1.45	45.15 ± 1.68	44.88 ± 1.84	0.565
ADG (g/d)	20.60 ± 1.20	21.14 ± 1.47	22.81 ± 1.56	21.15 ± 1.65	0.324
FCR	1.82 ± 0.40	2.16 ± 0.51	2.91 ± 0.64	2.21 ± 0.45	0.652
F/G	4.25 ± 0.51	4.58 ± 0.64	4.76 ± 0.72	4.81 ± 0.81	0.761
15–21 d					
BWG (g/d)	210.61 ± 5.81 ^c^	239.80 ± 10.35 ^b^	240.28 ± 8.08 ^b^	244.72 ± 9.28 ^a^	0.043
ADFI (g/d)	55.00 ± 2.14 ^c^	57.34 ± 2.61 ^b^	57.51± 2.86 ^b^	59.01 ± 2.65 ^a^	0.036
ADG (g/d)	26.19 ± 1.50 ^c^	28.25 ± 1.64 ^b^	28.15 ± 1.89 ^b^	29.59 ± 1.90 ^a^	0.045
FCR	2.76 ± 0.35	2.80 ± 0.43	2.64 ± 0.79	2.58 ± 0.49	0.751
F/G	4.30 ± 0.91	4.73 ± 0.71	4.78 ± 0.95	4.86 ± 1.00	0.684
22–28 d					
BWG (g/d)	280.04 ± 17.83 ^c^	311.40 ± 15.36 ^ab^	344.42 ± 10.97 ^b^	345.90 ± 15.18 ^a^	0.048
ADFI (g/d)	63.40 ± 2.10 ^b^	65.78 ± 2.56 ^ab^	71.05 ± 2.89 ^b^	71.95 ± 2.93 ^a^	0.043
ADG (g/d)	31.92 ± 1.94 ^c^	32.25 ± 1.98 ^ab^	35.45 ± 1.85 ^b^	35.77 ± 1.87 ^a^	0.044
FCR	2.81 ± 0.64	2.51 ± 0.70	2.60 ± 0.96	2.26 ± 0.92	0.360
F/G	4.36 ± 1.09	4.78 ± 0.99	4.80 ± 1.01	4.88 ± 0.94	0.428

^a–c^ Means with the same a row bearing different superscripts differ significantly (*p* < 0.05), MOLP-L: low dose of *Moringa oleifera* leaf polysaccharide, MOLP-M: medium dose of *Moringa oleifera* leaf polysaccharide, and MOLP-H: high dose of *Moringa oleifera* leaf polysaccharide.

**Table 3:** Effect of MOLP supplementation on organ indices (bursa, spleen, and liver) of broiler chickens at different developmental stages (0–14, 15–21, and 22–28 days).

Groups					
Organs	Control	MOLP-L (0.1 g/kg)	MOLP-M (0.2 g/kg)	MOLP-H (0.4 g/kg)	*p*-Value
0–14 d					
Bursa	0.734 ± 0.074	0.529 ± 0.046	0.554 ± 0.073	0.618 ± 0.118	0.271
Spleen	0.242 ± 0.413	0.167 ± 0.020	0.218 ± 0.028	0.184 ± 0.023	0.368
Liver	4.563 ± 0.464 ^c^	4.963 ± 0.179 ^b^	4.970 ± 0.234 ^b^	5.400 ± 0.421 ^a^	0.035
15–21 d					
Bursa	0.940 ± 0.122	0.976 ± 0.224	1.273 ± 0.378	1.249 ± 0.158	0.246
Spleen	0.335 ± 0.047	0.282 ± 0.025	0.335 ± 0.041	0.313 ± 0.071	0.310
Liver	6.188 ± 0.154	7.481 ± 0.736	8.166 ± 1.602	6.462 ± 0.450	0.425
22–28 d					
Bursa	1.382 ± 0.204 ^c^	1.653 ± 0.163 ^b^	1.636 ± 0.157 ^b^	1.936 ± 0.228 ^a^	0.041
Spleen	0.388 ± 0.020	0.425 ± 0.036	0.470 ± 0.043	0.400 ± 0.014	0.384
Liver	7.251 ± 0.588	7.602 ± 0.222	7.477 ± 0.442	6.026 ± 0.498	0.469

^a–c^ Means in the same a row bearing different superscripts differ significantly (*p* < 0.05), MOLP-L: low dose of *Moringa oleifera* leaf polysaccharide, MOLP-M: medium dose of *Moringa oleifera* leaf polysaccharide, and MOLP-H: high dose of *Moringa oleifera* leaf polysaccharide.

**Table 4:** The effect of MOLP on blood biochemical analysis.

Groups					
Items	Control	MOLP-L (0.1 g/kg)	MOLP-M (0.2 g/kg)	MOLP-H (0.4 g/kg)	*p*-Value
0–14 d					
TP (g/L)	39.8 ± 2.14	42.0 ± 3.31	41.4 ± 6.41	42.2 ± 4.54	0.412
ALB (g/L)	14.6 ± 2.40	12.0 ± 1.74	12.7 ± 3.47	13.4 ± 2.78	0.632
GLO (g/L)	25.2 ± 3.82	28.0 ± 2.58	27.7 ± 4.65	28.8 ± 3.55	0.784
ALB/GLO	0.7 ± 0.17	0.3 ± 0.45	0.4 ± 0.61	0.5 ± 0.12	0.320
CREA (µmol/L)	22.0 ± 2.54	21.4 ± 2.18	21.6 ± 2.58	21.1 ± 1.92	0.456
15–21 d					
TP (g/L)	34.83 ± 2.65	33.83 ± 1.07	33.16 ± 0.75	32.00 ± 0.30	0.482
ALB (g/L)	14.63 ± 0.29	14.06 ± 0.56	14.53 ± 0.31	13.76 ± 0.24	0.546
GLO (g/L)	18.63 ± 0.46 ^c^	23.16 ± 0.76 ^b^	23.20 ± 2.53 ^b^	26.23 ± 0.53 ^a^	0.042
ALB/GLO	0.66 ± 0.06	0.73 ± 0.03	0.80 ± 0.00	0.76 ± 0.03	0.263
CREA (µmol/L)	13.06 ± 1.31	15.56 ± 0.99	14.06 ± 1.13	11.40 ± 0.95	0.379
22–28 d					
TP (g/L)	34.93 ± 2.02 ^c^	39.33 ± 1.08 ^b^	39.46 ± 6.27 ^b^	43.60 ± 0.50 ^a^	0.037
ALB (g/L)	14.83 ± 0.88	15.06 ± 0.31	15.50 ± 0.40	13.46 ± 0.53	0.658
GLO (g/L)	20.10 ± 1.76	18.26 ± 0.80	20.10 ± 0.78	20.00 ± 5.98	0.491
ALB/GLO	0.74 ± 0.07	0.83 ± 0.03	0.78 ± 0.04	0.57 ± 0.10	0.315
CREA (µmol/L)	18.40 ± 9.67	16.20 ± 3.48	16.16 ± 1.96	17.46 ± 3.21	0.439

^a–c^ Means in the same a row bearing different superscripts differ significantly (*p* < 0.05), MOLP-L: low dose of *Moringa oleifera* leaf polysaccharide, MOLP-M: medium dose of *Moringa oleifera* leaf polysaccharide, and MOLP-H: high dose of *Moringa oleifera* leaf polysaccharide.

**Table 5:** Effects of MOLP supplementation on serum antioxidant indices of broiler chickens.

Groups					
Items	Control	MOLP-L (0.1 g/kg)	MOLP-M (0.2 g/kg)	MOLP-H (0.4 g/kg)	*p*-Value
0–14 d					
T-SOD (U/mL)	85.86 ± 2.14	85.92 ± 3.31	85.10 ± 3.41	85.10 ± 3.54	0.415
GSH-Px (U/mL)	223.18 ± 2.40	223.12 ± 1.74	223.72 ± 3.47	223.72 ± 2.78	0.647
T-AOC (μmol Trolox/mL)	1.08 ± 0.82	1.08 ± 0.58	1.09± 0.65	1.09 ± 0.55	0.770
MDA (nmol/mL)	6.33 ± 0.17	6.35 ± 0.45	6.33 ± 0.61	6.25 ± 0.12	0.348
15–21 d					
T-SOD (U/mL)	88.20 ± 2.50	88.45 ± 2.75	88.85 ± 3.01	88.96 ± 3.97	0.465
GSH-Px (U/mL)	230.42 ± 3.52	230.55 ± 3.74	230.85 ± 3.80	230.92 ± 3.88	0.560
T-AOC (μmol Trolox/mL)	1.18 ± 0.25	1.18 ± 0.14	1.18± 0.10	1.18 ± 0.09	0.652
MDA (nmol/mL)	7.13 ± 1.01	7.24 ± 1.23	7.52 ± 1.52	7.74 ± 1.84	0.571
22–28 d					
T-SOD (U/mL)	90.79 ± 3.41 ^c^	110.00 ± 23.30 ^b^	110.05 ± 3.85 ^b^	115.94 ± 3.87 ^a^	0.034
GSH-Px (U/mL)	240.21 ± 3.15 ^c^	311.80 ± 3.22 ^b^	312.15 ± 3.65 ^b^	345.45 ± 3.05 ^a^	0.048
T-AOC (μmol Trolox/mL)	1.27 ± 0.41 ^c^	1.53 ± 0.52 ^b^	1.50 ± 0.34 ^b^	1.79 ± 0.21 ^a^	0.041
MDA (nmol/mL)	7.28 ± 0.27 ^a^	6.91 ± 0.18 ^b^	6.88 ± 0.13 ^b^	6.46 ± 0.42 ^c^	0.037

^a–c^ Means in the same a row bearing different superscripts differ significantly (*p* < 0.05), MOLP-L: low dose of *Moringa oleifera* leaf polysaccharide, MOLP-M: medium dose of *Moringa oleifera* leaf polysaccharide, and MOLP-H: high dose of *Moringa oleifera* leaf polysaccharide.

## Data Availability

The original contributions presented in this study are included in the article. Further inquiries can be directed to the corresponding authors.
